# Whole genome sequencing reveals the impact of recent artificial selection on red sea bream reared in fish farms

**DOI:** 10.1038/s41598-019-42988-z

**Published:** 2019-04-24

**Authors:** Bo-Hye Nam, DongAhn Yoo, Young-Ok Kim, Jung Youn Park, Younhee Shin, Ga-hee Shin, Chan-Il Park, Heebal Kim, Woori Kwak

**Affiliations:** 1 Biotechnology Research Division, National Institute of Fisheries Science, Busan, 46083 Republic of Korea; 2 C&K genomics, 26 Beobwon-ro 9-gil H business Park Bldg. C, #1008, Songpa-gu, Seoul, Republic of Korea; 3 Interdisciplinary Program in Bioinformatics, Seoul National University, Seoul, Republic of Korea; 4 Research and Development Center, Insilicogen Inc., Gyeonggi-do, 16954 Republic of Korea; 5 Department of Marine Biology & Aquaculture, Gyeongsang National University, Tongyeong, 53064 Republic of Korea; 6 Department of Agricultural Biotechnology and Research Institute of Agriculture and Life Sciences, 7 Seoul National University, Seoul, Republic of Korea

**Keywords:** Agricultural genetics, Agricultural genetics, Conservation genomics, Conservation genomics

## Abstract

Red sea bream, a popular fish resource in Korea and Japan, is being bred in fish farms of the two countries. It is hypothesized that the genomes of red sea bream are influenced by decades of artificial selection. This study investigates the impact of artificial selection on genomes of red sea bream. Whole genome sequencing was conducted for 40 samples of red sea bream either from Ehime, Nagasaki and Tongyeong fish farms or from the wild. Population stratification based on whole genome data was investigated and the genomic regions of fish farm populations under selection were identified using XP-EHH and relative nucleotide diversity. Gene ontology analysis revealed that different functions were enriched in different fish farms. In conclusion, this study highlights the difference between independently cultured red sea bream populations by showing that influence of artificial selection acted upon completely different genes related to different functions including metabolic and developmental processes.

## Introduction

Red sea bream, *Pagrus major*, is a valuable aquaculture fish in Korea and Japan. Being an important fish resource in the two countries, red sea bream has been bred in aquacultures since 1980s in Korea^1^ and 1960s in Japan^2^. The aquaculture production of red sea bream in 2016 was 33,876 tons and 67,200 tons in Korea and Japan, respectively. To optimize the productivity of red sea bream, breeding programs have been established in Japanese fish farms for selecting and breeding offspring with better productivity traits such as higher growth rate^2^. Although not as systematic as in Japanese aquacultures, artificial selection is also being practiced in Korean fish farm based on measurable phenotypic traits including the weight and growth rate. As a result, several studies reported that the growth rate of breeding lines in Korean and Japanese fish farms have significantly increased^1–3^. However, the improved productivity of the red sea bream comes with the cost of reduced genetic diversity. The issue on breeding programs and the reduced genetic diversity has been previously discussed in other studies^4,5^; however, these studies did not make in-depth discussion on the impact of artificial selection on red sea bream genomes. To elucidate further on genetics of red sea bream and its relations to the selected traits, further analysis based on whole genome population data is necessary.

Red sea bream in the fish farms are exposed to intense artificial selection, which creates specific signatures or so-called ‘selective sweep’ in population genomes. Provided there is a superior allele in a population, the frequency of the beneficial allele within the population increases rapidly. This also results in increased frequency of hitchhiking variants near the beneficial allele, creating selective sweep signature^6,7^. A number of statistics can be used to detect these signatures including cross-population extended haplotype (XP-EHH)^8^, nucleotide diversity and cross-population composite likelihood ratio (XP-CLR)^9^. Using combinations of these statistics may allow us to detect genomic regions under selection more reliably^10,11^.

In case of other species being cultured in aquaculture, previous studies on artificial selection revealed various signatures of selection. Tilapia which is native cichlid fish from Middle East and Africa, exists in various commercial lines due to breeding program^12^. Regarding this fish, a previous study performed whole genome sequencing on selection lines and wild-type samples and performed selective sweep analysis to detect many genes under selection related to growth, immune system, response to chemical, stress and drug^12^. Previous study investigating artificial selection in Asian seabass revealed selection signature in the *prolactin* gene and its association with the growth traits^13^. In case of channel catfish, another previous study performed selective sweep analysis and identified genes including *hypoxia inducible factor 1b* (HIF1b) and *ATP-binding cassette sub-family B member 5* (ABCB5) which are related to productivity traits^14^. As such, the genes under selection varies for different species and we expect these markers to be different in red sea bream as well. Apart from monitoring decreased genetic diversity, discovery of genes showing signatures of selection may also facilitate marker-assisted selection which can lead to further improvement in productivity in aquaculture.

The aim of this publication is to investigate population genomes of red sea bream from various fish farms in Korea and Japan, and to detect genomic regions with selective sweep signature in each fish farm. Red sea bream populations were collected from three different fish farms (1 Korean and 2 Japanese farms) and pair-wise comparisons were made against a wild-type population collected from Korea using the statistics mentioned above. To our knowledge, this is the first study to perform population analysis on whole genome sequencing data of red sea bream.

## Results

### Features of four different red sea bream populations

**Figure 1:**
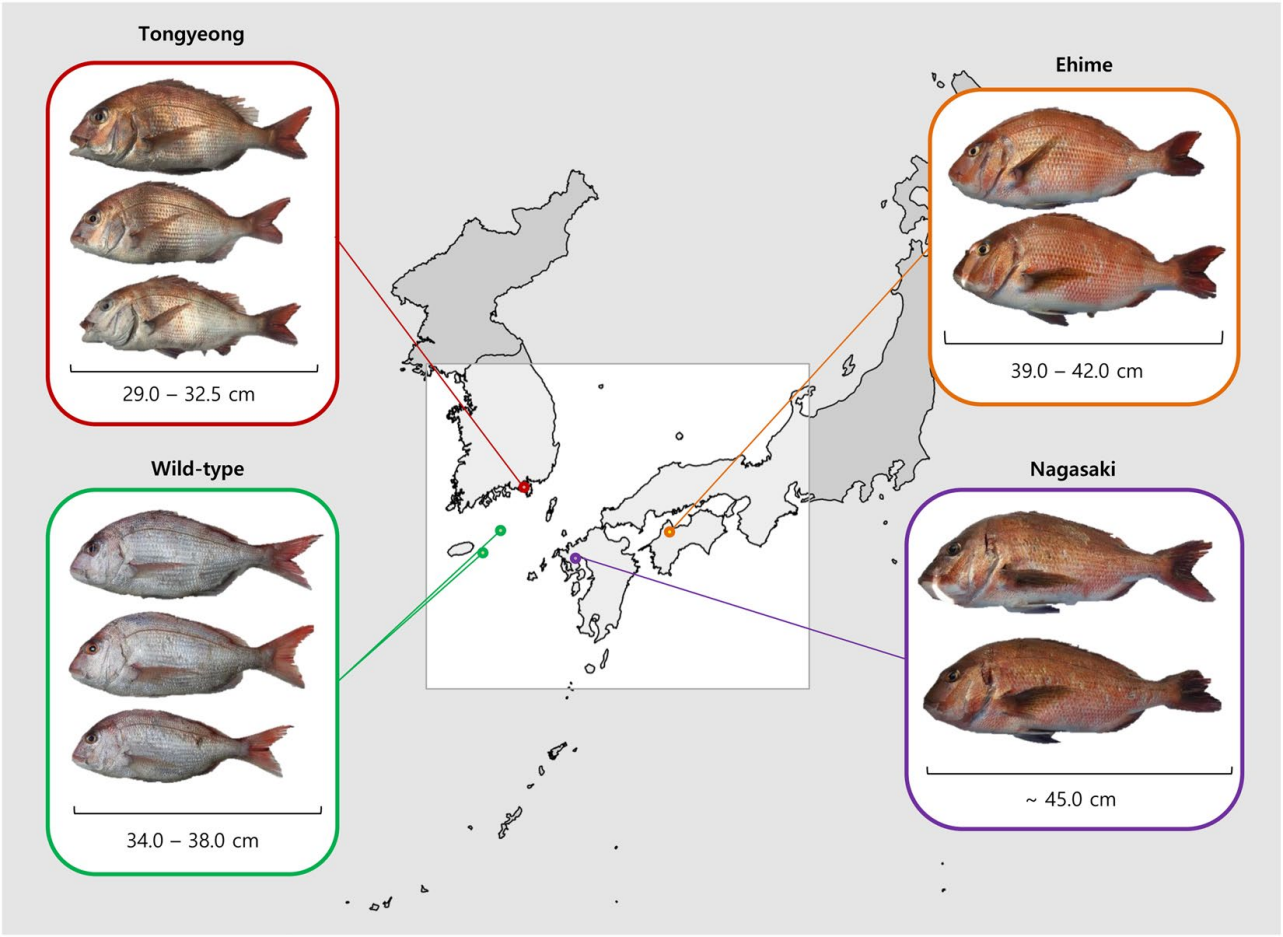
Red sea bream of Korea and Japan. The representative samples of red sea bream collected from Tongyeong, Ehime and Nagasaki fish farms and wild-type samples collected from two distinct locations of Jeju island, Korea are presented. (The map of Korea and Japan shown in Fig. 1 was drawn using R package “maps”)^30,31^.

The geographical location and the representative samples of each population were presented in Fig. 1. One major difference between the populations is the skin pigmentation which was assessed based on subjective visual inspection. The two Japanese populations show a characteristic red skin pigmentation. On the other hand, the colour of wild type population is much lighter than the Japanese populations. The colour of Tongyeong red sea bream is somewhere in between Japanese and the wild type. The body size of representative red sea bream samples were 38.5–42.0 cm, ~45.0 cm, 29.0–35.0 cm and 34.0–38.0 cm in Ehime, Nagasaki, Tongyeong and wild populations, respectively.

Red sea bream from Ehime fish farm composed of a strain developed by Marua Suisan (http://marua-suisan.co.jp/) with improved growth and disease resistance. The samples from Nagasaki fish farm originates from a strain developed by Nichimo marinculture (http://www.nichimo-mariculture.co.jp/) which also selected for fish with improved growth trait. Finally, the breeding strategy is again similar for Tongyeong population which selected for fish that are showing improved growth trait^1^. Comparison to the non-selected offspring of red sea bream showed that the developed line has higher weight gain and growth rate^1^.

In summary, different features of the red sea bream populations include skin pigmentation and body size. Also, note that the fish farm populations are composed of previously developed strains showing faster growth than the wild-types.

### Re-sequencing and identification of single nucleotide polymorphisms

The whole genome sequence data of 40 red sea breams were generated to ~10 × (~371Gbp), trimmed and aligned against Pagrus major 0.1 genome^15^, with average alignment rate of >90% (Additional File 1: Table S1–3). Variant calling on the population genome data resulted in discovery of 11,891,478 SNPs, contributing to 23,083,475 possible effects as predicted by SnpEff^16^ which annotates SNPs and predicts their effects in the red sea bream genome. The 9,348,913 predicted effects (40.4%) were identified for intergenic region. Many of the SNPs also were located upstream (19.0%), downstream (18.7%) of genes, or introns (9.4%). Among SNPs located in the protein coding region, 187,427 (0.811%) were synonymous variant while 102,402 (0.443%) were missense variant.

### Structure of different red sea bream populations

**Figure 2:**
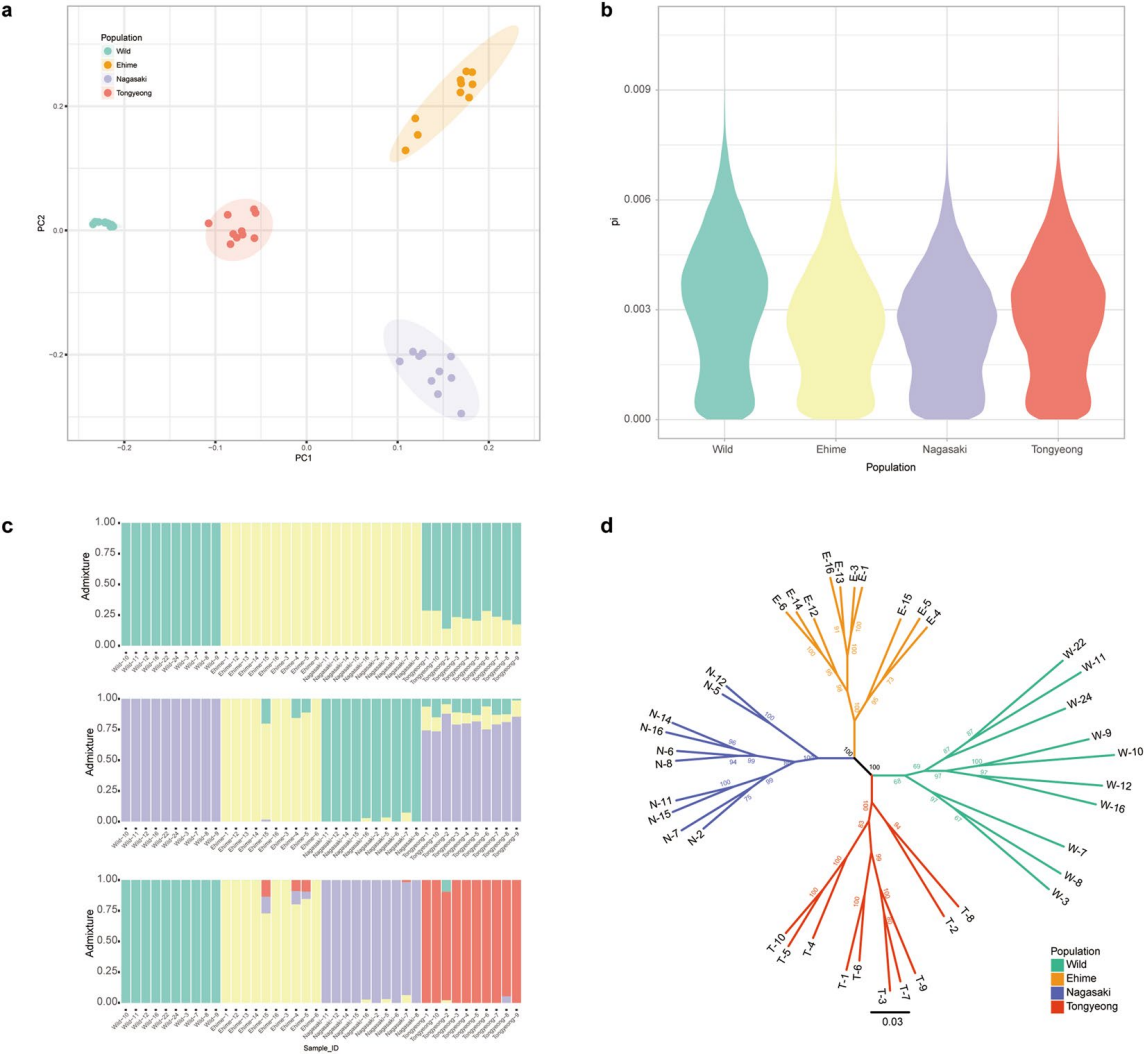
Population stratification analysis of red sea bream. The population stratification was visualized using (**a**) principal component analysis (PCA) based on genome-wide SNP data, (**b**) genome-wide nucleotide diversity distribution, (**c**) admixture analysis and (**d**) maximum likelihood tree.

Prior to discovery of the signature of artificial selection, population stratification of red sea bream was investigated based on the genome-wide SNP data. Principal component analysis (PCA) visualized the sample distribution in the two-dimension scatter plot of PC1 and PC2, without prior knowledge on the population. Four different clusters were observed. The red sea bream individuals from the same population were distributed in the same cluster without any exception (Fig. 2a). According to the PC1 and PC2, the wild-type samples collected from Korea were closer to one another than the rest of the populations (Fig. 2b).

The population genomic structure was investigated through admixture analysis with different number of ancestries, K ranging from 2 to 8 to determine the number of distinct ancestries of red sea bream population. As a result, we observed minimum cross-validation (CV) error at K = 2 (Additional file 1: Fig. S1), meaning the number of distinct ancestries of red sea bream population is the most likely to be 2. At K = 2, all wild-type red sea breams were distinguished from the individuals of the Japanese fish farms (Fig. 2c). The individuals from the Korean fish farm, Tongyeong were mostly composed of the same ancestry as the wild-type although they shared smaller portion with two Japanese farms. Similar observation was made at K = 3; however, as opposed to the previous result, majority of the genomic information between the two Japanese fish farms were not shared. At K = 4, we could observe stratification of the four populations.

Maximum likelihood phylogenetic tree was reconstructed based on the genome-wide SNP data. Again, clear distinction was observed between the four populations, aided by bootstrap value of 100% at the centre of the tree (Fig. 2d). Overall, combinations of different approaches for investigating population stratification produced unanimous results. The four different red sea bream populations were reproductively isolated and independently evolved to the extent that they are now distinguishable with SNP data.

### Selective sweep signature of red sea bream from three fish farms

Genomic regions of fish farm red sea bream under selection were investigated by identifying selective sweep signature. To detect such regions, independent pairwise comparison was performed against the wild type, using three statistics (XP-EHH, XP-CLR and relative nucleotide diversity). The regions with significantly high scores in only XP-EHH and relative nucleotide diversity statistics were considered as strong candidate regions; the result of XP-CLR could not be used due to extremely low XP-CLR statistics (Additional File 1: Fig. S2; Table S6).

**Figure 3:**
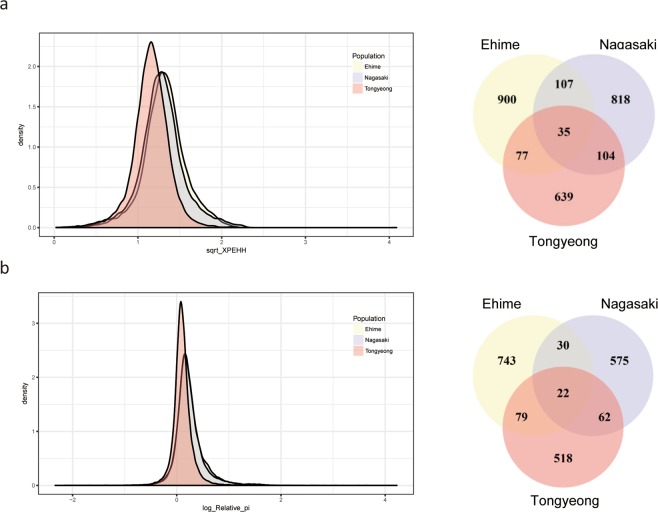
Selective sweep region of fish farm populations. The genome-wide distribution and the number of significant bins of (**a**) XP-EHH and (**b**) relative nucleotide diversity are presented.

XP-EHH was calculated over the genome-wide non-overlapping bins of 50 kb. Overall distribution of XP-EHH statistics was towards higher values in Ehime and Nagasaki populations compared to the Tongyeong population (Fig. 3a). The number of significant XP-EHH (p-value < 0.05) was also higher in the two Japanese populations (1,119 and 1,064, respectively) than that of Tongyeong population (855). Among the significant bins, 35 were found in all populations, while 900, 818 and 639 bins were specific to Ehime, Nagasaki and Tongyeong populations, respectively (Fig. 3a). To provide another evidence of selection, genome-wide nucleotide diversity was computed (Fig. 3b). As a result, 22 regions with significantly elevated values of relative nucleotide diversity (p-value < 0.05) were commonly identified in the three aquaculture populations (Ehime, Nagasaki and Tongyeong), while 743, 575 and 518 regions were specific to Ehime, Nagasaki and Tongyeong populations, respectively. Intersecting the results of XP-EHH and relative nucleotide diversity, the strong candidate regions of selection including 417, 462 and 253 bins in Ehime, Nagasaki and Tongyeong populations were identified (Table S5).

### Gene ontology term enrichment analysis of candidate genes under selection

**Figure 4:**
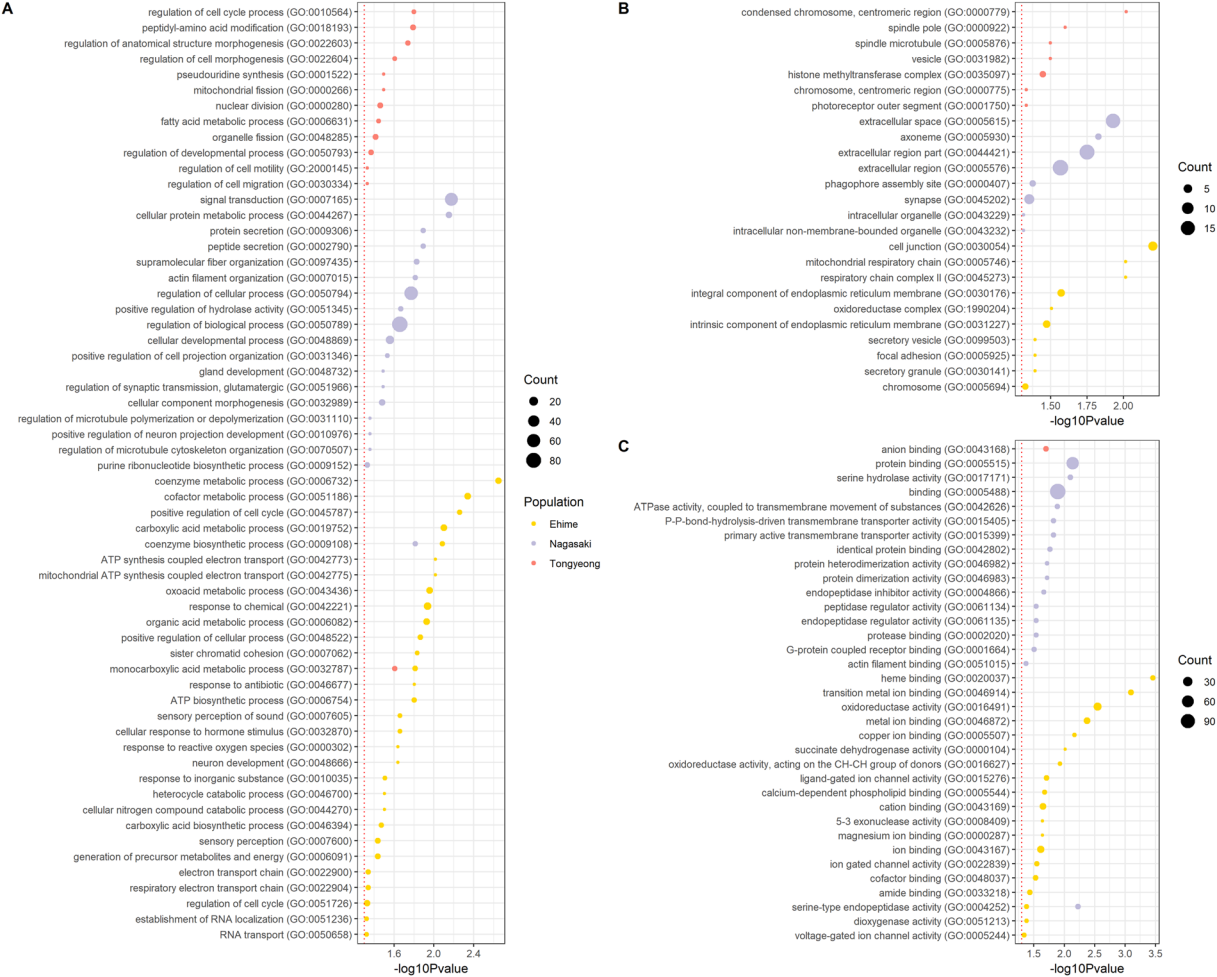
Functional annotation of candidate selective sweep genes. The summary of gene ontology (GO) analysis performed for (**A**) biological process, (**B**) cellular component and (**C**) molecular function in three fish farm populations are shown. Individual circle shows the significance of GO term and the size of the circle represents the number of genes associated with the GO term. The red dotted line represents p-value cut-off of 0.05.

To investigate the function of candidate regions under selection, we performed gene ontology (GO) analysis using Panther^17,18^ and DAVID^19,20^. We used 420, 549 and 325 genes of Ehime, Nagasaki and Tongyeong populations located in the candidate selective sweep regions (417, 462 and 253 bins) identified by intersecting XP-EHH and relative nucleotide diversity score. The significantly enriched GO terms identified using Panther are presented in Fig. 4. Interestingly, many GO terms related to metabolic processes were identified. Especially, various metabolic, catabolic and biosynthetic processes were found in Ehime population. Among these terms, “monocarboxylic acid metabolic process” and “coenzyme biosynthetic process” was shared with Tongyeong and Nagasaki populations, respectively (Fig. 4A). In addition, “fatty acid metabolic process” was found in Tongyeong and “cellular protein metabolic process” in Nagasaki populations. Consistent with this result, the Kyoto Encyclopedia of Genes and Genomes (KEGG) pathway from DAVID showed metabolic processes (Additional file 1: Fig. S3). Apart from metabolic processes, GO terms including development and morphogenesis were also detected (Fig. 4A). In Ehime, “neuron development” was found while “gland development”, “actin filament organization” and “positive regulation of neuron projection development” were found in Nagasaki. In Tongyeong, “regulation of developmental process” and “regulation of anatomical structure morphogenesis” were identified.

As a result of GO term analysis on cellular component, we found that the genes with selective sweep signature were associated with different cellular components in different fish farms (Fig. 4B). Similarly, the GO terms on molecular function were also completely different among fish farms (Fig. 4C).

### Identification of highly differentiating genotypes between fish farm populations

We define highly differentiating genotypes of a fish farm population as the genotypes that appear in significantly higher or lower frequency to that of wild-type population. For the three fish farm populations, pairwise comparison with the wild-type population was performed for the entire SNP loci to find highly differentiating genotypes at the respective positions. As a result, we identified 2,446,630, 2,325,736 and 1,622,269 loci with highly differentiating genotypes (p-value < 0.05) in Ehime, Nagasaki and Tongyeong populations, respectively. Comparing these loci with the candidate selective sweep regions (417, 462 and 253 bins of Ehime, Nagasaki and Tongyeong populations), we found 1,407, 420 and 1,133 overlapping loci in 43, 34 and 28 genes in Ehime, Nagasaki and Tongyeong populations, respectively (Table S7). Among overlapping genes, CASP7 was commonly identified in Ehime and Nagasaki, but we were unable to find any other common gene except for this gene.

## Discussion

Red sea bream has been bred in Korean and Japanese aquacultures since 1980s^1^ and 1960 s^2^. As an important fish resource in the two countries, many researches on red sea bream focused on improving its productivity or resistance to diseases; however, not as many genetic studies have been made on red sea bream. Among few previous studies, one study investigated genetic variability of inbred populations and showed that genetic variability has not dropped significantly^4^. On the other hand, another study admits that the genetic structure of red sea breams reared in captivity differ from the wild populations and further expressed concerns over genetic diversity of local populations perturbed by the large-scale hatchery releases^5^; however, these studies were limited to microsatellite markers and could not make in-depth discussion on the impact of breeding programs on red sea bream genomes. Not much is understood about what impacts decades of inbreeding might have had on genomes and phenomes of red sea bream. Further studies on whole genome data may provide deeper insight into the current status of red sea bream genomes and its relation to phenotypic traits. The current study analysed the red sea bream genomes based on whole genome population data. 40 red sea bream genomes collected from three different fish farms (Ehime, Nagasaki and Tongyeong) were investigated to reveal the population structure of fish farm populations and discover genes under selection.

Structure of the four different red sea bream populations were investigated by PCA, admixture and phylogenetic tree analysis. The population stratification was clearly observed in all analyses (Fig. 2), with decreased nucleotide diversity in the fish farm populations (Fig. 2b), which is possible outcome of breeding program selecting for fast-growing individuals. After observing that the four isolated populations are indeed distinguishable by some extent, selective sweep analysis was performed independently for each of the fish farm populations (Ehime, Nagasaki and Tongyeong). Here, it is important to note that wild-type population collected from Southern sea of Korea was used as the reference population, while the first generation in two Japanese fish farms were collected from different areas. Concerning this, a previous study reported that the red sea bream population collected from the sea ranging from Eastern sea from china to the sea nearby Japan showed high similarity^21^ as the fish can freely move along these areas. Consequently, selective sweep analysis was performed as we planned. The XP-EHH scores in the two Japanese populations were higher than that of Tongyeong (Fig. 3a). This can be due to higher degree of artificial selection in the Japanese populations, which is not surprising with Japanese populations given longer period for artificial selection than Korean population^1,2^. In agreement with the XP-EHH result, relative nucleotide diversity showed higher values in the two Japanese populations than Tongyeong population (Fig. 3b).

Based on the genes located in the selective sweep region that have significantly longer extension of haplotype and reduced nucleotide diversity, highly enriched GO terms were identified (Fig. 4). However, one limitation of this study, in identifying GO terms, was the use of the GO database for zebrafish due to the lack of a GO database for red sea bream. As mentioned in the result section, many GO terms related to metabolic processes were found and more importantly, at least one GO term related to metabolic process was identified in every fish farm population. This is an interesting observation as metabolic process is frequently observed in other farmed fish species selected for faster growth rate as well. In case of farmed Atlantic salmons which tend to have faster growth rate than the wild-types^22^, a previous study compared transcriptome profiles of farmed and wild Atlantic salmon and showed that the differentially expressed genes were highly associated with energy metabolism. This study also highlighted increased metabolic efficiency of farmed juvenile salmon compared to the wild-type^23^. Concerning the relationship between metabolism and growth, another previous study also showed that high standard metabolic rate could result in high efficiency in food intake needed for growth^24^. Similarly, selection for fast growing red sea bream population in Korean and Japanese fish farms^1,2^ could have resulted in selective sweep signatures near genes associated with metabolic pathways.

Apart from metabolic pathways, we also discovered GO terms related to developmental processes in the three fish farms. Among these terms, we found “actin filament organization” and “positive regulation of neuron projection development” in Nagasaki, and “regulation of developmental process” and “regulation of anatomical structure morphogenesis” in Tongyeong. This may be an evidence of artificial selection affecting developmental process or physical characteristics of farmed red sea bream which grows faster and tend to have higher body mass than the wild-type. Moreover, the GO terms related to development also include “neuron development” in Ehime and “gland development” and “positive regulation of neuron projection development” in Nagasaki population. This is an interesting observation as such functions are frequently observed in other domesticated species as well. According to a previous study, candidate regions under selection in domestic dogs were enriched for the GO term, central nervous system development^25^. Genes under selection found in another study for domestic pig^26^ and cattle^27^ were associated with nervous system. These studies view their genes as an evidence of artificial selection by relating them to behavioural traits of domesticated animals selected by human. Moving onto aquaculture for brown trout, it was reported that aquaculture altered behavioural traits of this fish^28^. Also, change of behavioural trait was also observed in domesticated strains of zebrafish^29^. Aquaculture might have resulted in similar outcome in red sea bream and thus, possible explanation for GO terms related to neuron development in red sea bream genomes can be the behavioural changes resulted from aquaculture.

In addition to GO term analysis, we have also investigated individual genes showing selective sweep signatures in different fish farms. Among them, 43, 34 and 28 genes in Ehime, Nagasaki and Tongyeong populations overlapped with SNP loci with highly differentiating genotypes compared to the wild population (Table S7). Surprisingly, almost all genes were not shared between different fish farm populations; only CASP7 was shared among Ehime and Nagasaki, a similar trend seen in GO term analysis. Apart from some of the metabolic processes, most of the highly enriched GO terms associated with biological process were not shared between fish farms; cellular components and molecular function were also completely different. This result highlights the difference between fish farm populations. As fish from these farms are cultured independently to one another, we predict that different parts of their genomes were affected by artificial selection.

Moving forward from the previous studies on genetic diversity of red sea bream, this study generated population genome data of red sea bream and performed population genome analysis to show the influence of artificial selection including stratified genomic structure. Through comparing the fish farm population to the wild-type population, we discovered that the genes under selection were different among fish farms although the breeding programs currently used in these fish farms aim for similar trait of growth. This means that artificial selection acted on different genomic regions in different fish farms along with independent breeding programs.

## Materials and Methods

### Sampling and sequencing of 40 red sea bream DNA

Prior to the sampling of wild type fish, permission was obtained from the National Institute of Fisheries Science committee under the IRB number 2017-NIFS-IACUC-08. 40 samples of red sea breams were used in this study, including 10 wild types from two distinct location near Jeju island, Korea and 30 breeding lines collected from 3 different fish farms (10 samples from Ehime and Nagasaki farms, Japan, and Tongyeong farm, Korea, respectively). The map of Korea and Japan shown in Fig. 1 was drawn using R package “maps”^30,31^.

The genomic DNA (~200 ng) from individual sample was extracted from the fin tissues and fragmented into smaller piece of ~500 bp, using Covaris M220 (Woburn, MA, USA). Indexed shotgun paired-end libraries for the fragmented DNA (~500 bp) were prepared, using TruSeq Nano DNA Library Prep Kit (Illumina, USA), following the standard Illumina sample-preparation protocol. For the genomic DNA, end repair, A-tailing and adapter ligation (~125 bp adapter) were performed followed by gel-based size selection for 550~650 bp and amplification with 8 cycles of PCR. Size distribution of the DNA was investigated by Agilent 2100 Bioanalyzer (Agilent Technologies). Finally, DNA sequencing was conducted using Illumina HiSeq2500 sequencer (2 × 150 bp paired-end reads were generated).

### Gene annotation of red sea bream genome

The genome assembly of Pagrus major 0.1 which was assembled in our previous study was used^15^. The function of protein coding genes in this genome was predicted by performing BLASTp alignment against Swiss-Prot database, three close fish genomes (*Tetraodon nigroviridis*, *Takifugu rubripes*, *Gasterosteus aculeatus*) and *Danio rerio* genome obtained from Ensembl database release-92^32,33^. BLAST hits with e-value < 10E-5 and bit score > 50 were used for the annotation, resulting in annotation of 21,341 genes (Table S4). With the same BLASTp alignment options, the gene annotation was also compared with Ensembl human and mouse genomes.

### Re-sequencing and variant discovery of red sea bream genomes

Base quality of sequenced data was visualized using fastQC prior to re-sequencing analysis (http://www.bioinformatics.bbsrc.ac.uk/projects/fastqc/). The adapter, low quality base (Q score < 20), read sequences with length < 75b and unpaired read sequences were removed via Trimmomatic-0.36^34^. Remaining high quality reads were aligned against the red sea bream reference genome Pagrus major 0.1, using Bowtie2-2.3.3.1^35^. The mapped reads were grouped and duplicated reads were filtered out using Picard 2.9.2 (http://picard.sourceforge.net). Local realignment was performed to correct errors caused by insertions and deletions (indels) using Genome-AnalysisTK-3.3-0 (GATK)^36^. Unifiedgenotyper of GATK was used to discover variants. Criteria used for the variant calling include: 1) count of reads that have mapped quality 0 (MQ0) < 4 and (MQ0/DP) = < 0.1, 2) variant call confidence (QUAL) = > 30, 3) QUAL score normalized by allele depth (QD) = > 5.0 and 4) strand bias score from Fisher’s Exact test (FS) = < 200. The high quality SNPs identified were used to recalibrate base quality. The second round of variant calling was conducted with the recalibrated data resulting in 11,891,478 SNPs and 2,150,618 indels. The effect of each SNP was predicted using SnpEff^16^. Imputation of the missing genotypes and phasing were performed using beagle 4.1^37^.

### Population structure of red sea bream

Population structure was examined based on the genome-wide SNP data. Principal component analysis (PCA) implemented in SNPRelate package^38^ was performed to visualize the sample distribution in a two-dimensional scatter plot. Additionally, admixture analysis was conducted for individual sample. Linkage disequilibrium (LD) effect was mitigated by incorporating filtering step. SNPs with pair-wise R2 values > 0.1 with any other SNPs within sliding window of 50 SNPs were filtered out using plink^39^, resulting in 996,004 SNPs. The ancestry fractions of individual red sea bream samples were estimated using ADMIXTURE^40^ with K ranging from 2 to 8 and bootstrap of 1,000. Maximum likelihood phylogenetic tree was reconstructed based on the genome-wide SNP data. SNPhylo software package^41^ was used with the bootstrap of 1,000. FigTree program was used for visualization of the tree.

### Identification of selective sweep region of red sea breams from fish farm

Different methods (XP-EHH, XP-CLR and relative nucleotide diversity) were used to identify selective sweep regions. XP-EHH compares the extended haplotypes between two populations^8^. Whereas, XP-CLR measures multi-locus allele frequency differentiation between two populations^9^. According to a previous study, both XP-EHH and XP-CLR can show high performance in case when there is low migration between two populations and XP-CLR is generally one of the best performing methods among the methods being compared^42^. Finally, relative nucleotide diversity compares the nucleotide diversity between two different populations. The three methods measure different signals (extended haplotype, allele frequency differentiation and diversity of the sequence) to find strong candidates of selective sweep.

For each of the 3 fish farm populations, pair-wise comparison against Korean wild type population was made using XP-EHH^8^ statistics (http://hgdp.uchicago.edu/Software/) implemented in SELSCAN^43^. The red sea bream genome was divided into 50 kb bin, and for each bin, the highest XP-EHH score was used as the representative score. Based on the observed score, normalized score was calculated, from which empirical p-value was computed. Empirical p-value of XP-EHH score assigned for each bin <0.05 was considered significant. The nucleotide diversity was calculated across non-overlapping 50 kb bins using VCFtools^44^. The relative nucleotide diversity was calculated as (pi _wild type_)/(pi _fish farms_). Note that higher value of relative nucleotide diversity represents reduced nucleotide diversity in the fish farm population. Similar to XP-EHH, empirical p-value of relative nucleotide diversity <0.05 was considered significant.

Additionally, pair-wise comparison of composite likelihood was performed using the source code for XP-CLR statistics available in (http://genetics.med.harvard.edu/reich/Reich_Lab/Software.html). The XP-CLR statistics was calculated across the non-overlapping bin of 50 kb. The maximum number of SNPs allowed within each bin was 600. The two SNPs with correlation level (R2) > 0.95 were down-weighted to prevent use of redundant SNPs during computation of composite likelihood^9^. The top 1% XP-CLR value were considered as the signature of selective sweep.

### Gene ontology term enrichment analysis of candidate genes located in selective sweep region

The closest genes were used to represent genomic regions identified in the selective sweep analysis. For the robustness of the GO analysis, genes with subject and query coverage of above 70% were used. GO analysis of the selective sweep region was performed by Panther^17,18^ and DAVID^19,20^, using the red sea bream genes as background and zebrafish GO database. GO terms with p-value < 0.05 were considered.

### Genotype-differentiation of fish farm populations

Genotype-differentiation of fish farm populations was identified using snpSift^45^. Assuming different genetic models including dominant, recessive, codominant, allelic and Cochran-Armitage trend models, a 2-by-2 or 2-by-3 contingency table was constructed with the number of observed genotypes in wild-type and fish farm populations; dominant, recessive and allelic models would require 2-by-2 contingency table while codominant and Cochran-Armitage trend models need 2-by-3 contingency table. Then, Fisher exact test (dominant, recessive and allelic models), chi-square test (codominant model) or Cochran-Armitage test (Cochran-Armitage model) was performed to identify highly differentiating genotypes of the fish farm populations compared to the wild-type. SNPs located in the genic regions, upstream or downstream of within 5 kb from the genes, showing p-value of below 0.05 under at least one of the models were considered as highly differentiating genotypes of the respective fish farm population. The polymorphic loci across the regions near the highly differentiating genotypes were visualized using ComplexHeatmap^46^.

## Supplementary information

Additional file 1

S4

S5

S5-2

S5-3

S6

S7
