# Author Correction: Whole genome sequencing reveals the impact of recent artificial selection on red sea bream reared in fish farms

**DOI:** 10.1038/s41598-020-58614-2

**Published:** 2020-01-28

**Authors:** Bo-Hye Nam, DongAhn Yoo, Young-Ok Kim, Jung Youn Park, Younhee Shin, Ga-hee Shin, Chan-Il Park, Heebal Kim, Woori Kwak

**Affiliations:** 1 Biotechnology Research Division, National Institute of Fisheries Science, Busan, 46083 Republic of Korea; 2 C&K genomics, 26 Beobwon-ro 9-gil H business Park Bldg. C, #1008, Songpa-gu, Seoul Republic of Korea; 3 Interdisciplinary Program in Bioinformatics, Seoul National University, Seoul, Republic of Korea; 4 Research and Development Center, Insilicogen Inc., Gyeonggi-do, 16954 Republic of Korea; 5 Department of Marine Biology & Aquaculture, Gyeongsang National University, Tongyeong, 53064 Republic of Korea; 6 Department of Agricultural Biotechnology and Research Institute of Agriculture and Life Sciences, 7 Seoul National University, Seoul, Republic of Korea

Correction to: *Scientific Reports* 10.1038/s41598-019-42988-z, published online 24 April 2019

In this Article, the Data availability section was omitted from the Additional Information section:

Availability of Data in NCBI Database: The DNA read data from the fish samples were submitted to the NCBI database under BioProject ID of PRJNA578498.

