# Supplementary material for: Whole genome sequencing reveals the impact of recent artificial selection on red sea bream reared in fish farms
Source: Sci Rep. 2019 Apr 24;9:6487. doi: 10.1038/s41598-019-42988-z (PMC6482192; doi:10.1038/s41598-019-42988-z)
Supplement: Supplementary file 1 — Additional file 1 [file 41598_2019_42988_MOESM1_ESM.docx]

**Supplementary Information**

**Whole genome sequencing reveals the impact of recent artificial selection on red sea bream reared in fish farms**

Bo-Hye Nam^1¶^, DongAhn Yoo^3¶^, Young-Ok Kim^1^, Jung Youn Park^1^, Younhee Shin^4^, Ga-hee Shin^4^, Chan-Il Park^5^, Heebal Kim^2,3,6^, Woori Kwak^2^

^1^Biotechnology Research Division, National Institute of Fisheries Science, Busan 46083, Republic of Korea

^2^C&K genomics, 26 Beobwon-ro 9-gil H business Park Bldg. C, #1008, Songpa-gu, Seoul, Republic of Korea

^3^Interdisciplinary Program in Bioinformatics, Seoul National University, Seoul, Republic of Korea

^4^Research and Development Center, Insilicogen Inc., Gyeonggi-do 16954, Republic of Korea

^5^Department of Marine Biology & Aquaculture, Gyeongsang National University, Tongyeong 53064, Republic of Korea

^6^Department of Agricultural Biotechnology and Research Institute of Agriculture and Life Sciences, 7

Seoul National University, Seoul, Republic of Korea.

¶These authors contributed equally to this work.

*Corresponding author E-mail: asleo@cnkgenomics.com

**
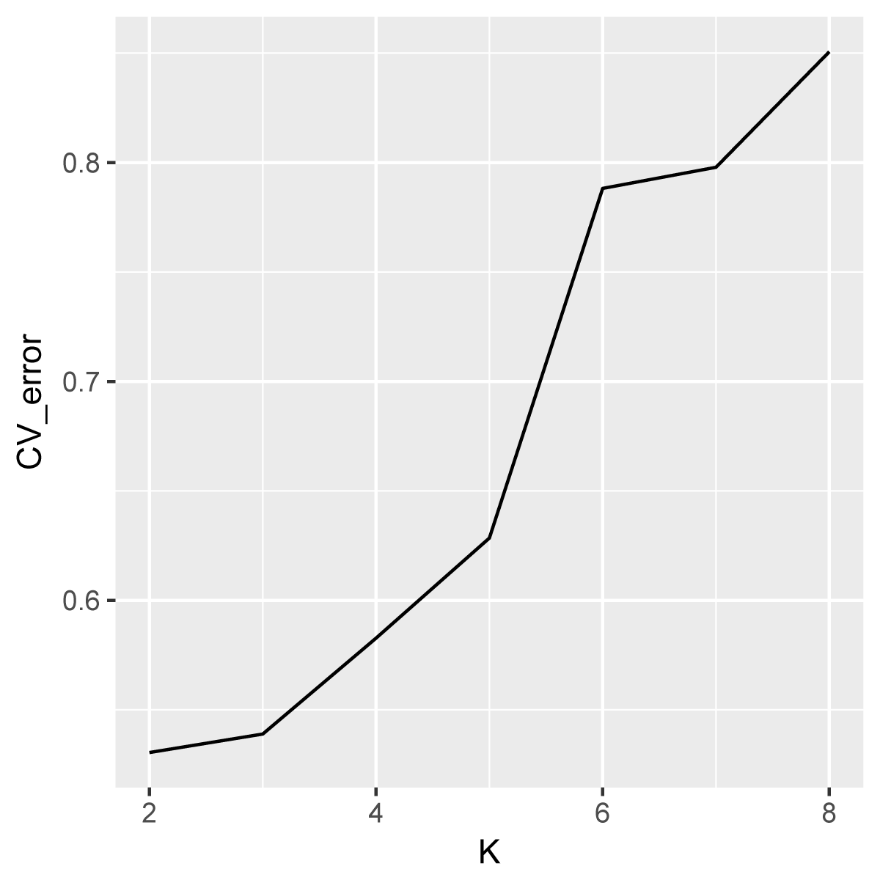
**

**Supplementary S1 Fig. Cross validation error of admixture analysis.**

**
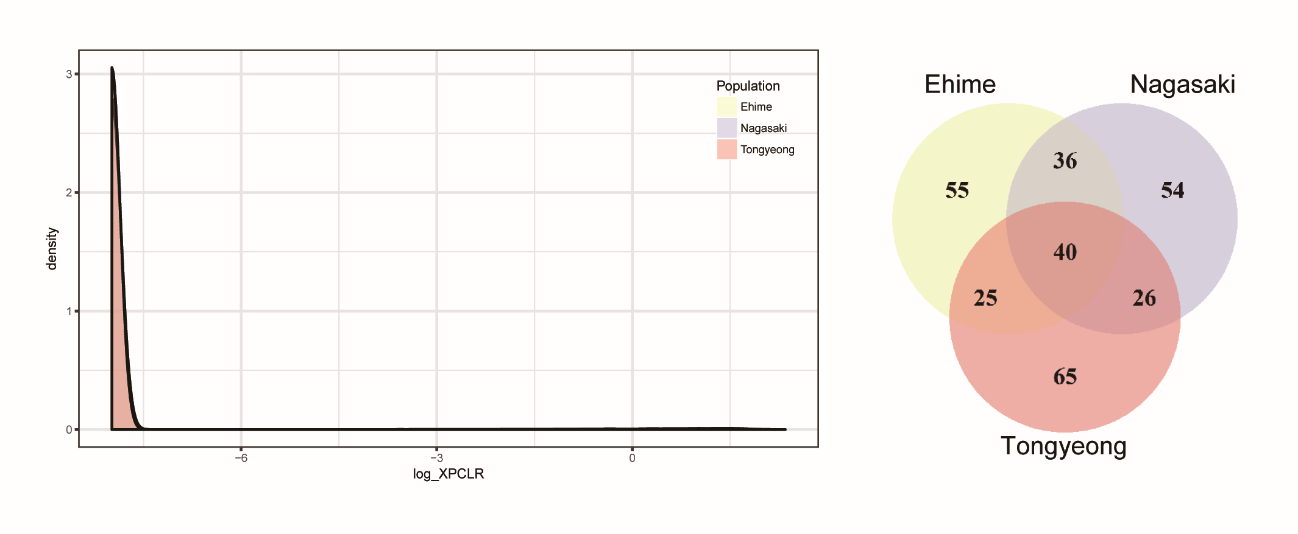
**

**Supplementary Fig. S2. Selective sweep region of fish farm populations.** The genome-wide distribution and the number of significant bins of XP-CLR are presented.


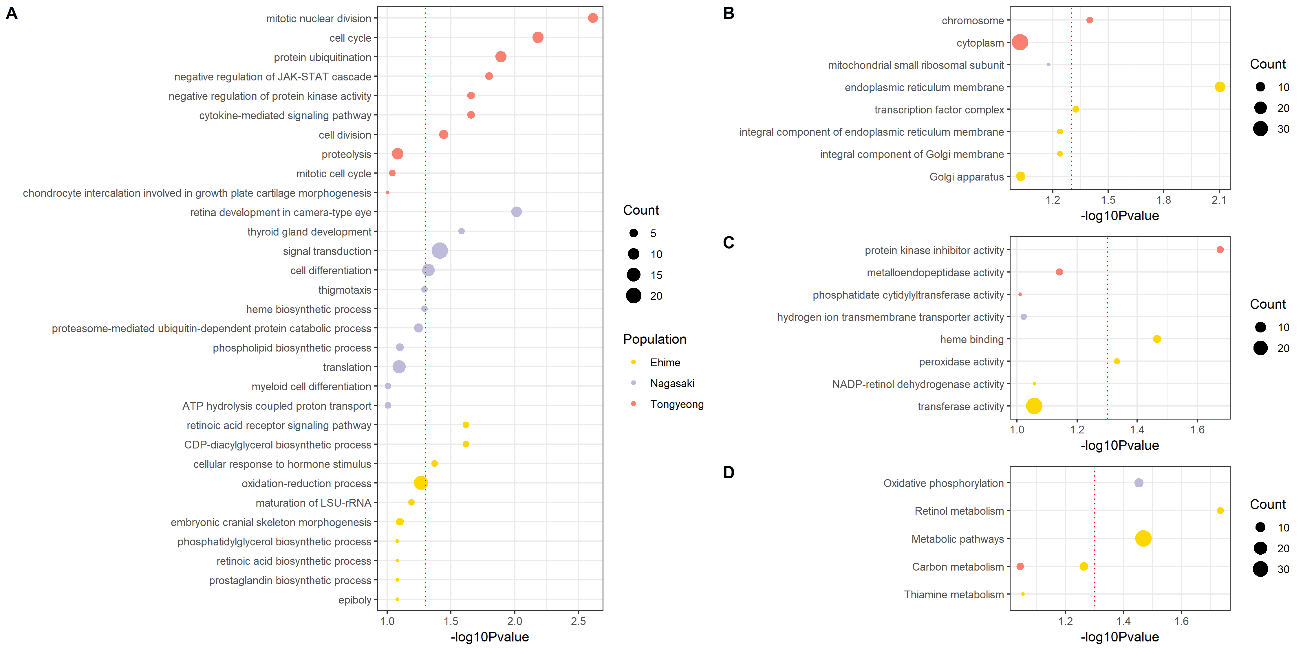


**Supplementary Fig. S3. Functional annotation of candidate selective sweep genes.** The summary of gene ontology (GO) analysis performed for (a) biological process, (b) cellular component, (c) molecular function and (d) Kyoto Encyclopedia of Genes and Genomes (KEGG) pathway in three fish farm populations are shown. Individual circle shows the significance of GO term and the size of the circle represents the number of genes associated with the GO term. The red dotted line represents p-value cut-off of 0.05.

**Supplementary Table S1. Raw data summary**

| **Sample name** | **Read length** | **Read count** | **GC** | **Q20 ratio** | **Q30 ratio** |
| --- | --- | --- | --- | --- | --- |
| **Domestics-03** | 151 | 30421144 | 41 | 0.99837 | 0.914 |
| **Domestics-07** | 151 | 32031622 | 41 | 0.99841 | 0.915 |
| **Domestics-08** | 151 | 30442442 | 41 | 0.99843 | 0.92 |
| **Domestics-09** | 151 | 31557397 | 41 | 0.99846 | 0.916 |
| **Domestics-10** | 151 | 33619489 | 41 | 0.99846 | 0.917 |
| **Domestics-11** | 151 | 33532579 | 41 | 0.99853 | 0.916 |
| **Domestics-12** | 151 | 33576688 | 41 | 0.99846 | 0.916 |
| **Domestics-16** | 151 | 30618472 | 41 | 0.9984 | 0.916 |
| **Domestics-22** | 151 | 28489832 | 41 | 0.99844 | 0.917 |
| **Domestics-24** | 151 | 31481729 | 41 | 0.99845 | 0.918 |
| **Ehime-01** | 151 | 30511646 | 41 | 0.99881 | 0.863 |
| **Ehime-03** | 151 | 25846114 | 41 | 0.99885 | 0.856 |
| **Ehime-04** | 151 | 31560299 | 41 | 0.99879 | 0.861 |
| **Ehime-05** | 151 | 30742432 | 41 | 0.99878 | 0.863 |
| **Ehime-06** | 151 | 27896334 | 41 | 0.99857 | 0.883 |
| **Ehime-12** | 151 | 29489225 | 41 | 0.99868 | 0.883 |
| **Ehime-13** | 151 | 33061772 | 40 | 0.99851 | 0.882 |
| **Ehime-14** | 151 | 30777923 | 41 | 0.99852 | 0.88 |
| **Ehime-15** | 151 | 31596118 | 41 | 0.9985 | 0.879 |
| **Ehime-16** | 151 | 32298680 | 41 | 0.99862 | 0.878 |
| **Nagasaki-02** | 151 | 32086808 | 41 | 0.9987 | 0.861 |
| **Nagasaki-05** | 151 | 28665731 | 41 | 0.9988 | 0.858 |
| **Nagasaki-06** | 151 | 31905369 | 41 | 0.99873 | 0.858 |
| **Nagasaki-07** | 151 | 29103059 | 41 | 0.99875 | 0.855 |
| **Nagasaki-08** | 151 | 29899486 | 41 | 0.99873 | 0.857 |
| **Nagasaki-11** | 151 | 28645118 | 41 | 0.99868 | 0.859 |
| **Nagasaki-12** | 151 | 29815876 | 41 | 0.99877 | 0.86 |
| **Nagasaki-14** | 151 | 29412073 | 41 | 0.99879 | 0.861 |
| **Nagasaki-15** | 151 | 25533229 | 41 | 0.99879 | 0.863 |
| **Nagasaki-16** | 151 | 30140236 | 41 | 0.9988 | 0.86 |
| **Tongyeong-01** | 151 | 31401125 | 41 | 0.99849 | 0.881 |
| **Tongyeong-02** | 151 | 31821619 | 41 | 0.99842 | 0.883 |
| **Tongyeong-03** | 151 | 32041371 | 41 | 0.99847 | 0.882 |
| **Tongyeong-04** | 151 | 29749697 | 40 | 0.99854 | 0.884 |
| **Tongyeong-05** | 151 | 32995551 | 41 | 0.99827 | 0.877 |
| **Tongyeong-06** | 151 | 32030688 | 41 | 0.99837 | 0.876 |
| **Tongyeong-07** | 151 | 30508115 | 41 | 0.9984 | 0.879 |
| **Tongyeong-08** | 151 | 30845774 | 41 | 0.99852 | 0.875 |
| **Tongyeong-09** | 151 | 31226714 | 41 | 0.99825 | 0.914 |
| **Tongyeong-10** | 151 | 31243441 | 41 | 0.99825 | 0.916 |

**Supplementary Table S2. Data trimming summary**

| **Sample**  **name** | **Total**  **reads** | **Both surviving** | **Forward only**  **surviving** | **Reverse only**  **surviving** | **Drop** |
| --- | --- | --- | --- | --- | --- |
| **Domestics-03** | 30421144 | 30003490 (98.63%) | 301207 (0.99%) | 5308 (0.02%) | 111139 (0.37%) |
| **Domestics-07** | 32031622 | 31569885 (98.56%) | 336803 (1.05%) | 7847 (0.02%) | 117087 (0.37%) |
| **Domestics-08** | 30442442 | 30043516 (98.69%) | 292541 (0.96%) | 5361 (0.02%) | 101024 (0.33%) |
| **Domestics-09** | 31557397 | 31111145 (98.59%) | 327785 (1.04%) | 5386 (0.02%) | 113081 (0.36%) |
| **Domestics-10** | 33619489 | 33124833 (98.53%) | 362342 (1.08%) | 5935 (0.02%) | 126379 (0.38%) |
| **Domestics-11** | 33532579 | 33057820 (98.58%) | 357052 (1.06%) | 5621 (0.02%) | 112086 (0.33%) |
| **Domestics-12** | 33576688 | 33147978 (98.72%) | 310956 (0.93%) | 6093 (0.02%) | 111661 (0.33%) |
| **Domestics-16** | 30618472 | 30182379 (98.58%) | 312428 (1.02%) | 7527 (0.02%) | 116138 (0.38%) |
| **Domestics-22** | 28489832 | 28122905 (98.71%) | 265310 (0.93%) | 6831 (0.02%) | 94786 (0.33%) |
| **Domestics-24** | 31481729 | 31067229 (98.68%) | 312026 (0.99%) | 5536 (0.02%) | 96938 (0.31%) |
| **Ehime-01** | 30511646 | 29962907 (98.20%) | 342486 (1.12%) | 3515 (0.01%) | 202738 (0.66%) |
| **Ehime-03** | 25846114 | 25388718 (98.23%) | 289468 (1.12%) | 2778 (0.01%) | 165150 (0.64%) |
| **Ehime-04** | 31560299 | 31047245 (98.37%) | 323092 (1.02%) | 3397 (0.01%) | 186565 (0.59%) |
| **Ehime-05** | 30742432 | 30215609 (98.29%) | 327867 (1.07%) | 4339 (0.01%) | 194617 (0.63%) |
| **Ehime-06** | 27896334 | 27474509 (98.49%) | 271888 (0.97%) | 3714 (0.01%) | 146223 (0.52%) |
| **Ehime-12** | 29489225 | 29029543 (98.44%) | 299042 (1.01%) | 3939 (0.01%) | 156701 (0.53%) |
| **Ehime-13** | 33061772 | 32544950 (98.44%) | 327921 (0.99%) | 4492 (0.01%) | 184409 (0.56%) |
| **Ehime-14** | 30777923 | 30304905 (98.46%) | 302778 (0.98%) | 3657 (0.01%) | 166583 (0.54%) |
| **Ehime-15** | 31596118 | 31051400 (98.28%) | 341092 (1.08%) | 4769 (0.02%) | 198857 (0.63%) |
| **Ehime-16** | 32298680 | 31782359 (98.40%) | 335818 (1.04%) | 4121 (0.01%) | 176382 (0.55%) |
| **Nagasaki-02** | 32086808 | 31543620 (98.31%) | 334241 (1.04%) | 3549 (0.01%) | 205398 (0.64%) |
| **Nagasaki-05** | 28665731 | 28115562 (98.08%) | 343004 (1.20%) | 3464 (0.01%) | 203701 (0.71%) |
| **Nagasaki-06** | 31905369 | 31298877 (98.10%) | 371317 (1.16%) | 3693 (0.01%) | 231482 (0.73%) |
| **Nagasaki-07** | 29103059 | 28644931 (98.43%) | 287024 (0.99%) | 2899 (0.01%) | 168205 (0.58%) |
| **Nagasaki-08** | 29899486 | 29383071 (98.27%) | 319918 (1.07%) | 4212 (0.01%) | 192285 (0.64%) |
| **Nagasaki-11** | 28645118 | 28157415 (98.30%) | 300058 (1.05%) | 4373 (0.02%) | 183272 (0.64%) |
| **Nagasaki-12** | 29815876 | 29312371 (98.31%) | 314561 (1.06%) | 2708 (0.01%) | 186236 (0.62%) |
| **Nagasaki-14** | 29412073 | 28922506 (98.34%) | 304087 (1.03%) | 3077 (0.01%) | 182403 (0.62%) |
| **Nagasaki-15** | 25533229 | 25156772 (98.53%) | 233362 (0.91%) | 2718 (0.01%) | 140377 (0.55%) |
| **Nagasaki-16** | 30140236 | 29620001 (98.27%) | 326076 (1.08%) | 3139 (0.01%) | 191020 (0.63%) |
| **Tongyeong-01** | 31401125 | 30952033 (98.57%) | 287306 (0.91%) | 3841 (0.01%) | 157945 (0.50%) |
| **Tongyeong-02** | 31821619 | 31385618 (98.63%) | 276363 (0.87%) | 4620 (0.01%) | 155018 (0.49%) |
| **Tongyeong-03** | 32041371 | 31605522 (98.64%) | 277781 (0.87%) | 3844 (0.01%) | 154224 (0.48%) |
| **Tongyeong-04** | 29749697 | 29355327 (98.67%) | 256149 (0.86%) | 4521 (0.02%) | 133700 (0.45%) |
| **Tongyeong-05** | 32995551 | 32582582 (98.75%) | 260050 (0.79%) | 4117 (0.01%) | 148802 (0.45%) |
| **Tongyeong-06** | 32030688 | 31603611 (98.67%) | 270646 (0.84%) | 3800 (0.01%) | 152631 (0.48%) |
| **Tongyeong-07** | 30508115 | 30091832 (98.64%) | 262696 (0.86%) | 3760 (0.01%) | 149827 (0.49%) |
| **Tongyeong-08** | 30845774 | 30415892 (98.61%) | 276504 (0.90%) | 3550 (0.01%) | 149828 (0.49%) |
| **Tongyeong-09** | 31226714 | 30899967 (98.95%) | 232811 (0.75%) | 5366 (0.02%) | 88570 (0.28%) |
| **Tongyeong-10** | 31243441 | 30916121 (98.95%) | 231396 (0.74%) | 6005 (0.02%) | 89919 (0.29%) |

**Supplementary Table S3. Alignment summary**

| **Sample name** | **Overall alignment rate** | **Concordant**  **zero** | **Concordant pair**  **alignment** | **Multiple**  **alignment** |
| --- | --- | --- | --- | --- |
| **Domestics-03** | 94.32% | 21.91% | 63.84% | 14.25% |
| **Domestics-07** | 94.39% | 20.27% | 65.30% | 14.42% |
| **Domestics-08** | 92.95% | 23.20% | 62.94% | 13.85% |
| **Domestics-09** | 93.85% | 22.48% | 63.53% | 13.98% |
| **Domestics-10** | 92.43% | 24.31% | 61.83% | 13.86% |
| **Domestics-11** | 93.42% | 22.46% | 63.41% | 14.13% |
| **Domestics-12** | 93.78% | 22.40% | 63.41% | 14.19% |
| **Domestics-16** | 92.87% | 23.51% | 62.54% | 13.96% |
| **Domestics-22** | 93.82% | 22.32% | 63.48% | 14.20% |
| **Domestics-24** | 94.25% | 21.63% | 63.92% | 14.44% |
| **Ehime-01** | 93.37% | 23.03% | 63.82% | 13.14% |
| **Ehime-03** | 93.32% | 22.58% | 63.91% | 13.51% |
| **Ehime-04** | 93.40% | 22.77% | 63.67% | 13.56% |
| **Ehime-05** | 93.43% | 22.87% | 63.84% | 13.29% |
| **Ehime-06** | 94.00% | 23.05% | 63.31% | 13.64% |
| **Ehime-12** | 94.02% | 24.51% | 62.41% | 13.08% |
| **Ehime-13** | 93.92% | 24.88% | 62.12% | 13.00% |
| **Ehime-14** | 93.89% | 25.30% | 61.69% | 13.00% |
| **Ehime-15** | 93.81% | 25.29% | 61.49% | 13.22% |
| **Ehime-16** | 93.85% | 25.40% | 61.52% | 13.08% |
| **Nagasaki-02** | 93.42% | 22.38% | 63.87% | 13.75% |
| **Nagasaki-05** | 93.29% | 23.97% | 62.93% | 13.10% |
| **Nagasaki-06** | 93.31% | 23.48% | 63.08% | 13.44% |
| **Nagasaki-07** | 93.25% | 22.89% | 63.67% | 13.44% |
| **Nagasaki-08** | 93.34% | 23.54% | 63.15% | 13.32% |
| **Nagasaki-11** | 93.33% | 23.86% | 62.77% | 13.37% |
| **Nagasaki-12** | 93.45% | 23.29% | 63.49% | 13.22% |
| **Nagasaki-14** | 93.41% | 22.12% | 64.47% | 13.41% |
| **Nagasaki-15** | 93.44% | 23.43% | 63.30% | 13.27% |
| **Nagasaki-16** | 93.32% | 23.69% | 63.13% | 13.18% |
| **Tongyeong-01** | 93.84% | 23.91% | 62.29% | 13.79% |
| **Tongyeong-02** | 93.76% | 24.72% | 62.07% | 13.22% |
| **Tongyeong-03** | 93.86% | 24.75% | 61.97% | 13.28% |
| **Tongyeong-04** | 93.88% | 23.34% | 63.14% | 13.53% |
| **Tongyeong-05** | 93.57% | 25.21% | 61.14% | 13.66% |
| **Tongyeong-06** | 93.61% | 24.97% | 61.51% | 13.51% |
| **Tongyeong-07** | 93.72% | 23.80% | 62.49% | 13.71% |
| **Tongyeong-08** | 93.69% | 23.72% | 62.74% | 13.54% |
| **Tongyeong-09** | 94.62% | 25.64% | 60.89% | 13.47% |
| **Tongyeong-10** | 94.68% | 25.00% | 61.80% | 13.19% |
